# No Overall Survival Difference in the Immunotherapy Era for Rare Subtypes of Melanoma

**DOI:** 10.3389/pore.2021.639004

**Published:** 2021-06-17

**Authors:** Mohammed Safi, Dario Trapani, Mohammed Alradhi, Xiu Shan, Liu Jiwei

**Affiliations:** ^1^ Department of Oncology, The First Affiliated Hospital of Dalian Medical University, Dalian, China; ^2^ Istituto Europeo di Oncologia (IEO), Milan, Italy; ^3^ Department of Urology, The Second Affiliated Hospital of Dalian Medical University, Dalian, China

**Keywords:** melanoma, SEER database, immunotherapy, survival, epidimiology

We read with interest the article by Uprety et al. on the melanoma survival between contemporary periods depending on the approval time of immunotherapy [1]. I want to congratulate the authors for this fruitful article and make some contributions.

In the study, it has been indicated that the immunotherapy era was significantly added benefits to overall survival (OS) for melanoma patients; however, the rare sites of melanoma should be included in these two contemporary groups. Unluckily, the patients with these relatively rare melanoma subtypes (acral lentiginous, uveal, and mucosal melanomas) which typically do not respond to the emerging immunotherapy that has been approved for the more common type of melanoma, and thus have worse overall survival rates [2] and attempting to reach enduring safe and effective responses in these high-risk subtypes of melanoma is one of the field's main challenges [3].

**FIGURE 1:**
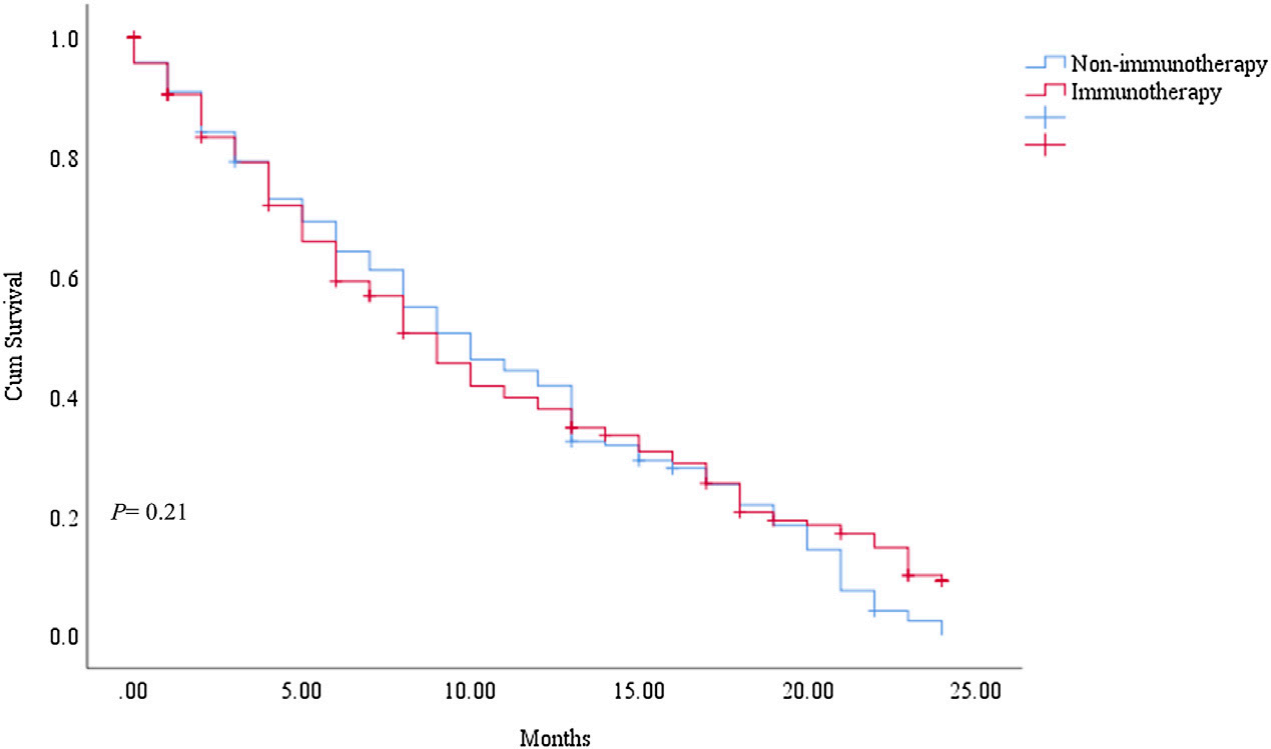
Kaplan Meier–OS difference between rare subtypes of melanoma (*p* = 0.31). 1- Immunotherapy era. 2- non-immunotherapy era.

We searched for the distant rare subtypes of melanoma (Stage - 6th edition. Derived AJCC M, 6th ed (2004-2015) from Epidemiology, and End Results (SEER) Program (www.seer.cancer.gov) SEER*Stat Database 3.8.9 version: Incidence - SEER Research Data, 18 Registries, Nov 2019 Sub (2000-2017), and follow-up. We revealed that there was no significant difference in survival between immunotherapy and non-immunotherapy era *p* = 0.31. Figure 1 Our findings give more attention to the clinician in practice with the rare melanoma subtypes in the era of immunotherapy.

We strongly highlight effective clinical and preclinical studies toward these rare subtypes of melanoma, including the combination of immunotherapy and anti-vascular agents (NCT03955354, NCT03991975, NCT03602547), new immune checkpoint inhibitors (NCT02071940 with an anti-CSF1) and cell-based approaches (NCT01983748) [3, 4].
